# Stereoselective and Simultaneous Analysis of Ginsenosides from Ginseng Berry Extract in Rat Plasma by UPLC-MS/MS: Application to a Pharmacokinetic Study of Ginseng Berry Extract

**DOI:** 10.3390/molecules23071835

**Published:** 2018-07-23

**Authors:** Seong Yon Han, Min Goo Bae, Young Hee Choi

**Affiliations:** College of Pharmacy and Research Institute for Drug Development, Dongguk University_Seoul, 32 Dongguk-lo, Ilsandong-gu, Goyang, Gyeonggi-do 10326, Korea; hsyglory@gmail.com (S.Y.H.); nophra88@naver.com (M.G.B.)

**Keywords:** ginseng berry extract, ginsenosides, stereoselective and simultaneous analysis, pharmacokinetics, oral administration

## Abstract

The role of ginseng berry extract (GBE) has been attributed to its anti-hyperglycemic effect in humans. However, the pharmacokinetic characteristics of GBE constitutes after oral GBE administration have not been established yet. In this study, stereoselective and simultaneous analytical methods for 10 ginsenosides (ginsenoside Rb1, Rb2, Rc, Rd, Re, Rg1, S-Rg2, R-Rg2, S-Rg3, and R-Rg3) were developed using ultra-performance liquid chromatography, coupled with electrospray ionization triple quadrupole tandem mass spectrometry (UPLC-MS/MS), for the pharmacokinetic study of GBE. Furthermore, the pharmacokinetic profiles of 10 ginsenosides after oral GBE were evaluated in rats. All analytes were detected with a linear concentration range of 0.01–10 µg/mL. Lower limits of detection (LLOD) and quantification (LLOQ) were 0.003 and 0.01 µg/mL, respectively, for all 10 ginsenosides. This established method was adequately validated in linearity, sensitivity, intra- and inter-day precision, accuracy, recovery, matrix effect, and stability. Relative standard deviations for all intra- and inter-precision of the 10 ginsenosides were below 11.5% and accuracies were 85.3–111%, which were sufficient to evaluate the pharmacokinetic study of oral GBE in rats. We propose that Rb1, Rb2, Rc, Rd, Re, Rg1, S-Rg2, R-Rg2 and/or S-Rg3 were appropriate pharmacokinetic markers of systemic exposure following oral GBE administration.

## 1. Introduction

The recent focus on ginseng berry (GB), fruit of *Panax ginseng* C.A. Meyer, is attributed to its pharmacological activities against atherosclerosis, diabetic mellitus, obesity, inflammation, allergy, and systemic lupus erythematosus [1,2,3,4,5,6,7]. Furthermore, the anti-hyperglycemic effect of GB extract was recently reported [8].

In most ginseng species, ginsenosides are the major active constituents and responsible for various pharmacological activities [9]. GB also contains various ginsenosides related to the pharmacological properties of GB [10,11,12,13,14,15,16]. However, the composition of ginsenosides in GB is distinctly different from that of ginseng roots [13,17,18,19]. The GB extract (GBE) used in this study contains a higher level of ginsenoside Re with more potent anti-hyperglycemic effect of GBE compared to ginseng root extract in preclinical and clinical trials [8,20]. Furthermore, the structural isomerism of ginsenosides has been reported to contribute to multiple pharmacological effects [21].

One or more active constituents can hardly be attributed to herb extract. The pharmacokinetic properties of constituents contained in an herb extract vary from that of isolated components [22,23,24,25,26,27,28]. The co-existing constituents show different pharmacokinetic profiles in terms of absorption, distribution, metabolism, and excretion of active constituent(s), which interfere with the efficacy and safety of an herbal mixture [24,25]. Based on this perspective, pharmacokinetic studies of multi-components in an herb extract are essential for understanding the pharmacological effects and therapeutic efficacy of an herb extract. Thus, it is necessary to evaluate the pharmacokinetic profiles of various ginsenosides following oral administration of GBE. Although pharmacokinetic reports of ginsenosides exist [20,29,30], there is no simultaneous and steroselective analytical method to evaluate the pharmacokinetics of ginsenosides following oral administration of GBE. Moreover, a different part (stems-leaves of *Panax ginseng* [27]) and processed ginseng (red ginseng [30]) were used and the analytical method was developed only in rat urine [15]. The pharmacokinetic profile of ginsenoside Re alone in GBE was investigated [20] in previous reports.

**Figure 1:**
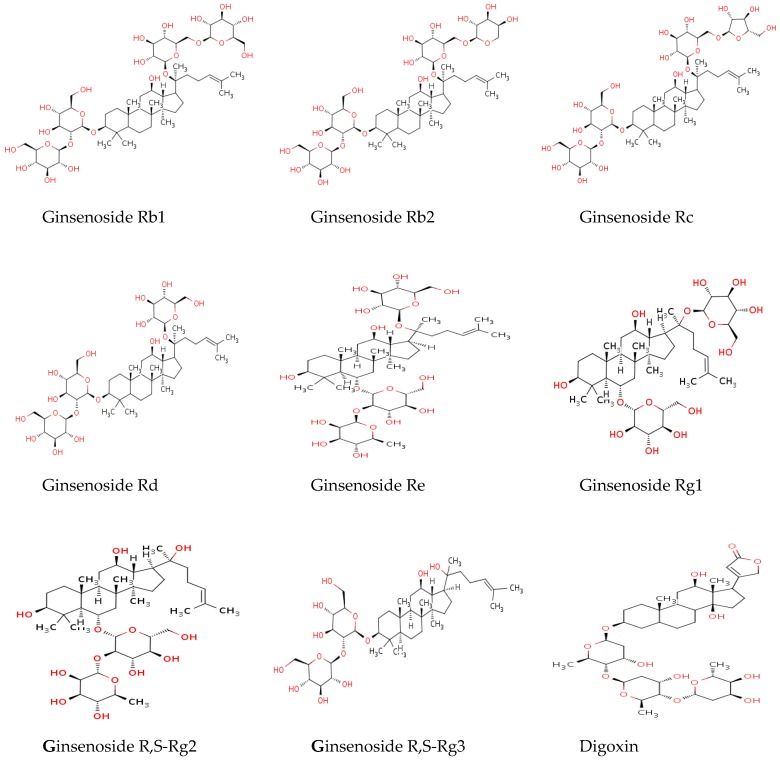
Chemical structures of ginsenosides and digoxin (IS). Ginsenosides Rb1, Rb2, Rc, Rd, R-Rg3, and S-Rg3 are protopanaxadiol (PPD)-type ginsenosides. Also ginsenosides Re, Rg1, R-Rg2 and S-Rg2 are PPT-type ginsenosides.

In the present study, we selected 10 active constituents (Rb1, Rb2, Rc, Rd, Re, Rg1, S-Rg2, R-Rg2, S-Rg3, and R-Rg3) (Figure 1) of GBE based on the high content of ginsenosides in GBE. The simultaneous and stereoselective quantification of 10 ginsenosides in rat plasma using a fully validated accurate, rapid, and sensitive UPLC-MS/MS method was established. For the first time, the pharmacokinetic properties of 10 ginsenosides after oral administration of GBE were investigated, using this validated method.

## 2. Results

### 2.1. UPLC-MS/MS Method Validation

#### 2.1.1. Selectivity

**Figure 2:**
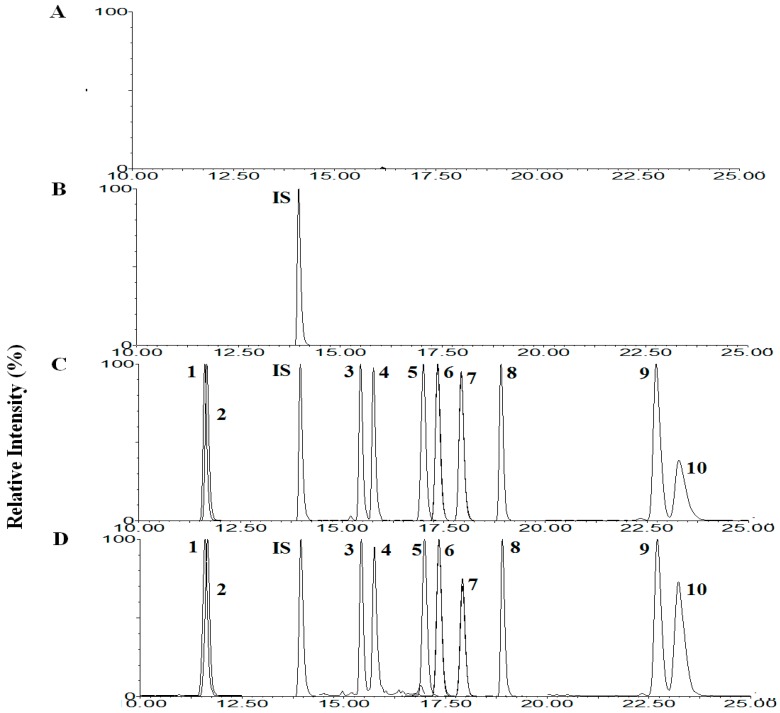

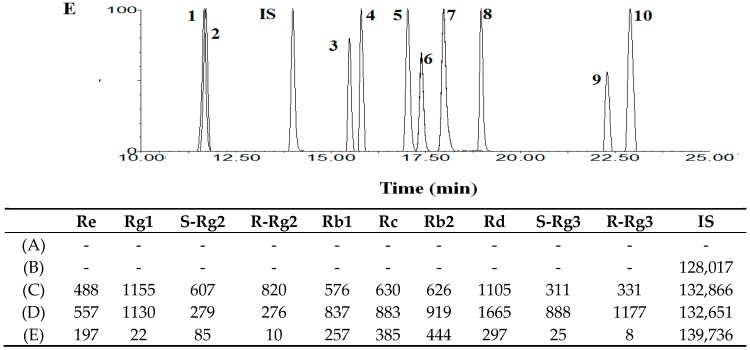
Mass chromatogram after deprotenization with methanol for double blank plasma (**A**), rat blank plasma with IS (**B**), stock solution of Rb1, Rb2, Rc, Rd, Re, Rg1, R-Rg2, S-Rg2, R-Rg3 and S-Rg3 (**C**), plasma spiked with 0.05 µg/mL of Rb1, Rb2, Rc, Rd, Re, Rg1, R-Rg2, S-Rg2, R-Rg3 and S-Rg3 (**D**), and a plasma sample from 5 min after oral administration of 600 mg (5 mL)/kg ginseng berry extract (GBE) in rats (**E**). Also the area values of each ginsenoside and IS are shown in the box. 1, Re; 2, Rg1; 3, S-Rg2; 4, R-Rg2; 5, Rb1; 6, Rc; 7, Rb2; 8, Rd; 9, S-Rg3; 10, R-Rg3.

There was no interfering peak from endogenous substrates at the elution times: Rb1, Rb2, Rc, Rd, Re, Rg1, R-Rg2, S-Rg2, R-Rg3, S-Rg3 and IS peak at 17.07, 18.01, 17.43, 19.00, 11.70, 11.75, 15.84, 15.51, 23.98, 22.94 and 14.04 min, respectively. Typical chromatograms for stock solution, drug-free rat plasma, spiked with 0.05 µg/mL of Rb1, Rb2, Rc, Rd, Re, Rg1, R-Rg2, S-Rg2, R-Rg3, S-Rg3 and a plasma sample after oral administration of 600 mg (5 mL)/kg GBE in rats are shown in Figure 2. The total run time per sample was 30 min, however, chromatograms in Figure 2 were detected from 10 to 30 min. The concentrations of Rb1, Rb2, Rc, Rd, Re, Rg1, R-Rg2, S-Rg2, R-Rg3, S-Rg3 in rat plasma at 90 min after the oral administration of GBE were 0.128, 0.165, 0.139, 0.251, 0.184, 0.024, 0.008, 0.005, 0.009 and 0.002 µg/mL, respectively.

#### 2.1.2. Linearity and Sensitivity

The calibration curves of Rb1, Rb2, Rc, Rd, Re, Rg1, R-Rg2, S-Rg2, R-Rg3, and S-Rg3 in rat plasma exhibited good linearity with correlation coefficient (*r*) within the range of 0.982 to 1.000 (Table 1). The lower limit of quantification (LLOQ) was 0.01 µg/mL for all ginsenosides.

#### 2.1.3. Precision and Accuracy

Intra- and inter-day precision and accuracy of the method were assessed by measuring LLOQ and four different QC samples on five different days. The results are shown in Table 2. The coefficients of variation (CVs) for intra- and inter-day precision were 4.86 and 4.09% for Rb1, 3.54 and 2.09% for Rb2, 11.0 and 5.28% for Rc, 6.99 and 10.2% for Rd, 3.41 and 14.9% for Re, 3.39 and 10.8% for Rg1, 4.91 and 3.47% for R-Rg2, 7.79 and 3.60% for S-Rg2, 11.5 and 9.07% for R-Rg3, and 10.8 and 10.4% for S-Rg3, respectively. The intra-(and inter-) day accuracies were 95.0–101 (97.0–100)% for Rb1, 98.7–101 (98.6–109)% for Rb2, 95.0–102 (98.1–111)% for Rc, 94.3–97.3 (95.1–98.9)% for Rd, 93.1–102 (86.0–100)% for Re, 93.9–102 (94.3–101)% for Rg1, 88.1–103 (88.3–103)% for R-Rg2, 89.7–102 (90.4–110)% for S-Rg2, 99.5–105 (89.3–99.8)% for R-Rg3, and 95.6–107 (97.6–101)% for S-Rg3, respectively. The QC samples were within 15% of the nominal concentrations, meeting the acceptance criteria of the US Food and Drug Administration (FDA) for the validation of bioanalytical methods [31].

#### 2.1.4. Matrix Effect

Three different QC samples and drug-free plasma were used to evaluate the effects of the sample matrix on the ionization of 10 ginsenosides. The percentages of the matrix effects of Rb1, Rb2, Rc, Rd, Re, Rg1, R-Rg2, S-Rg2, R-Rg3, and S-Rg3 at four different concentrations were 101%, 102%, 97.9%, 103%, 105%, 104%, 97.7%, 101%, 106%, and 104%, respectively.

#### 2.1.5. Stability

After confirming the stability of stock solution of each ginsenoside (at least 93% of each ginsenoside in stock solution remained for 1 week at 4 °C and −80 °C in our unpublished data), the stability test of ginsenosides in plasma was conducted. No significant degradation (within ±15% deviation between the predicted and nominal concentrations) of Rb1, Rb2, Rc, Re, Rg1, R-Rg2, S-Rg2, R-Rg3, and S-Rg3 occurred in rat plasma under the following conditions: short-term storage for 24 h at room temperature (25 °C), three times freeze-thaw cycles, post-treatment storage for 12 h at 4 °C, and long-term storage for 28 days at −80 °C (Table 3). In case of Rd, 72.9–78.4% of spiked concentration was recovered at post-treatment storage for 12 h at 4 °C, without any degradation under other conditions.

### 2.2. Pharmacokinetic Studies in Rats

**Figure 3:**
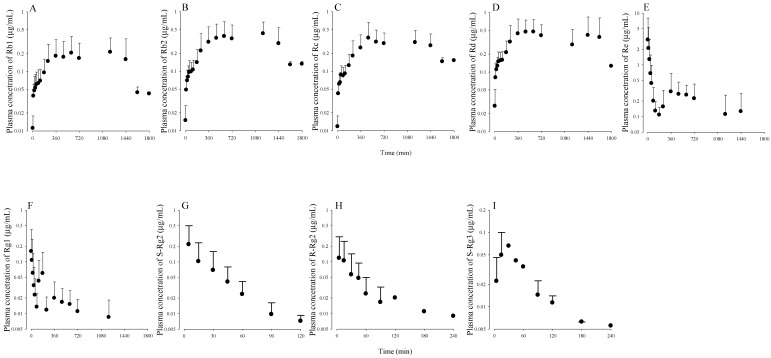
Plasma concentration of ginsenosides, (**A**) Rb1, (**B**) Rb2, (**C**) Rc, (**D**) Rd, (**E**) Re, (**F**) Rg1, (**G**) S-Rg2, (**H**) R-Rg2, and (**I**) S-Rg3, after oral administration of 600 mg/kg GBE to rat.

To evaluate the utility of the ultra-performance liquid chromatography, coupled with electrospray ionization triple quadrupole tandem mass spectrometry (UPLC-MS/MS) method developed, pharmacokinetic studies were conducted in rats after the oral administration of 600 mg/kg GBE. The mean arterial plasma concentration-time profiles of Rb1, Rb2, Rc, Re, Rg1, R-Rg2, S-Rg2, and R-Rg3 are shown in Figure 3. The relevant pharmacokinetic parameters are summarized in Table 4. 

The percentages of Rb1, Rb2, Rc, Rd, Re, Rg1, R-Rg2, S-Rg2, R-Rg3 and S-Rg3 content in GBE were 1.29, 2.56, 0.86, 3.31, 6.50, 0.24, 0.47, 0.75, 0.14, and 0.36%, respectively. In terms of dosage, 7.74, 15.4, 5.16, 19.9, 39.0, 1.44, 2.82, 4.50, 0.84, and 2.16 mg/kg of Rb1, Rb2, Rc, Rd, Re, Rg1, R-Rg2, S-Rg2, R-Rg3 and S-Rg3, respectively, were orally administered when 600 mg/kg of GBE was orally administered to rats in this study.

To compare the systemic exposure of Rb1, Rb2, Rc, Rd, Re, Rg1, R-Rg2, S-Rg2, and S-Rg3 based on AUC_last_ and *C*_max_ values, these parameters were normalized according to each dose administered as 1 mg/kg because of varying amounts of each ginsenoside, including GBE. The normalized AUC_last_ values of Rb1, Rb2, Rc, Rd, Re, Rg1, R-Rg2, S-Rg2 and S-Rg3 at 1 mg/kg were 26.1, 24.5, 68.5, 27.3, 8.96, 15.8, 1.32, 1.71 and 4.25 µg·min/mL at 1 mg/kg dose, respectively. The normalized AUC_last_ values of four protopanaxadiol (PPD)-type ginsenosides: Rb1, Rb2, Rc, and Rd, were much higher than those of PPT-type ginsenosides: Re, Rg1, S-Rg2, R-Rg2, and S-Rg3 (Table 4). The normalized *C*_max_ values of Rb1, Rb2, Rc, Rd, Re, Rg1, R-Rg2, S-Rg2, and S-Rg3 were 27.2, 25.7, 75.1, 26.1, 85.2, 15.6, 51.4, 44.3, and 56.0 ng/mL at 1 mg/kg dose, respectively.

## 3. Discussion

The analytical method developed for 10 ginsenosides in this study was adequate for the pharmacokinetic studies of GBE based on selectivity, linearity, sensitivity, precision, accuracy, matrix effect, and stability using UPLC-MS/MS. In the selectivity test, no interfering peak from endogenous substrates was detected at the elution times of Rb1, Rb2, Rc, Rd, Re, Rg1, R-Rg2, S-Rg2, R-Rg3, S-Rg3, and IS peaks. To separate the four isomers, the total running time was 30 min for each injection, which was relative for the separation of the isomers. The degree of separation met the analytical criteria without interference from the adjacent peaks. The calibration curves of the 10 ginsenosides in the ranges of 0.01–10 µg/mL showed good linearity and the sensitivity of each ginsenoside facilitated the pharmacokinetic study of Rb1, Rb2, Rc, Rd, Re, Rg1, R-Rg2, S-Rg2, R-Rg3, and S-Rg3 after oral administration of GBE in rats. The intra- and inter-day precision and accuracy of the method were in the acceptance range of the US FDA criteria for the validation of bioanalytical methods [31]. No significant matrix effects were detected for any analytes in the matrix effect test and no significant degradation except Rd in the condition of post-preparation at 25 °C was observed in the stability test. Based on this information for Rd, the prepared samples were kept at 4 °C in plate of auto-sampler plate in the UPLC-MS/MS system in order to accurately measure the concentration of Rd in the plasma sample.

Generally, bioactive constituents which exhibit favorable pharmacokinetic properties following substantial systemic exposure to the herb extract, are referred to as pharmacokinetic markers [32]. In particular, pharmacokinetic markers facilitate the evaluation of efficacy and toxicity as well as drug interactions based on the pharmacokinetic properties of bioactive constituents in an herbal extract. We measured rat plasma’s systemic exposure to six protopanaxadiol (PPD) and four protopanaxatriol (PPT) ginsenosides after oral administration of GBE. The sensitivity of the analytical method developed was sufficient to characterize the pharmacokinetics of nine of the ginsenosides, except R-Rg3. Although the level of S-Rg3 (0.84%) was lower than R-Rg3 (2.16%) in GBE, the plasma concentrations of S-Rg3 were detected but those of R-Rg3 were detected only at only certain time points, probably due to the different pharmacokinetic profiles of epimers such as the longer *T*_max_ of S-Rg3 compared with that of R-Rg3 (Table 4).

To predict the systemic exposure of Rb1, Rb2, Rc, Rd, Re, Rg1, R-Rg2, S-Rg2, and S-Rg3, their AUC_last_ and *C*_max_ values were normalized by each dose contained in GBE because the amount of each ginsenoside, including GBE, varied. Assuming that a dose of Rb1, Rb2, Rc, Rd, Re, Rg1, R-Rg2, S-Rg2, and S-Rg3 contained in GBE is in the range of linear pharmacokinetics, the normalized AUC_last_ values of Rb1, Rb2, Rc, Rd, Re, Rg1, R-Rg2, S-Rg2 and S-Rg3 at 1 mg/kg were 26.1, 24.5, 68.5, 27.3, 8.96, 15.8, 1.32, 1.71 and 4.25 µg·min/mL, respectively. The normalized AUC_last_ values of the four PPD-type ginsenosides Rb1, Rb2, Rc and Rd were much higher than those of PPT-type ginsenosides, Re, Rg1, S-Rg2, R-Rg2, and S-Rg3 (Table 4). Similar results were observed after oral administration of PPD or PPT-type ginsenosides as a single compound [30]. These data appear to indicate that the PPD-type ginsenosides (e.g., Rb1, Rb2, Rc, Rd) were absorbed better in the rat gastrointestinal tract than the PPT-type ginsenoside Re. It was also reported that the relatively low plasma concentrations of PPT-type ginsenosides might be due poor intestinal absorption of PPT-type ginsenosides [30,33]. The *T*_max_ values of PPD-type ginsenosides were lower than those of PPT-type ginsenosides, suggesting that the absorption rates of PPD-type ginsenoisdes were slower than those of PPT-type ginsenosides.

The normalized *C*_max_ values of Rb1, Rb2, Rc, Rd, Re, Rg1, R-Rg2, S-Rg2, and S-Rg3 were 27.2, 25.7, 75.1, 26.1, 85.2, 15.6, 51.4, 44.3, and 56.0 ng/mL at 1 mg/kg dose, respectively. In spite of the higher or similar normalized *C*_max_ values of PPT-type ginsenosides compared to those of PPD-type ginsenosides, the normalized AUC_last_ values of PPT-type ginsenosides were lower than those of PPD-type ginsenosides (Table 4), probably due to rapid biliary excretion of PPT-type ginsenosides as reported previously [33]. Interestingly, the secondary peaks of Re and Rg1 in plasma concentration profiles observed in our study (Figure 3) may be attributed to biliary excretion and enterohepatic circulation of Re and Rg1. 

The systemic exposure of PPD-type ginsenosides in rat plasma might possibly be elevated due to their concentrations in GBE, good solubility, and long t_1/2_ of approximately 10–20 h, based on the previous report [30]. However, S-Rg3 and R-Rg3 exhibited relatively lower systemic exposure with shorter t_1/2_, which might be related to rapid and extensive biliary excretion or other mechanisms [33]. Therefore, the pharmacokinetic study of GBE components is essential for our understanding of its pharmacological effects on the body.

In conclusion, the pharmacokinetic parameters of Rb1, Rb2, Rc, Rd, Re, Rg1, R-Rg2, S-Rg2, and S-Rg3 after oral administration of GBE to rats were successfully validated using analytical method described in this study for the first time. This method was selective, precise, accurate and reliable for the simultaneous determination of Rb1, Rb2, Rc, Rd, Re, Rg1, R-Rg2, S-Rg2, R-Rg3, and S-Rg3 in rat plasma using UPLC–MS/MS. The pharmacokinetic results of 10 ginsenosides in GBE showed that Rb1, Rb2, Rc, Rd, Re, Rg1, R-Rg2, and S-Rg2 levels in the plasma were appropriate pharmacokinetic markers of GBE in rats because of their high exposure levels. Most importantly, oral ingestion of ginsenosides from GBE yielded significantly higher ratios and slow rates of absorption of PPD-type ginsenosides (e.g., Rb1. Rb2, Rc and Rd), suggesting that ginsenoside structures facilitated the prediction of their pharmacokinetic profiles including absorption in herbal medicines. Therefore, the structural and pharmacological profiles may explain the efficacy and safety of GBE, warranting further clinical investigations.

## 4. Materials and Methods 

### 4.1. Chemicals and Reagents

Ginsenoside Rb1 (Rb1), ginsenoside Rb2 (Rb2), ginsenoside Rc (Rc), ginsenoside Rd (Rd), ginsenoside Re (Re), ginsenoside Rg1 (Rg1), ginsenoside R-Rg2 (R-Rg2), ginsenoside S-Rg2 (S-Rg2), ginsenoside R-Rg3 (R-Rg3), and ginsenoside S-Rg3 (S-Rg3) were purchased from Chengdu Bio-Purify Phytochemicals Ltd. (Sichuan, China). Digoxin [internal standard (IS) for ultra-performance liquid chromatography-tandem mass spectrometry (UPLC-MS/MS) analysis] was purchased from Sigma-Aldrich (St. Louis, MO, USA). All structures of ginsenosides and IS used in this study are displayed in Figure 1. All solvents of high-performance liquid chromatographic grade were purchased from Fisher Scientific Co. (Seoul, South Korea) and other chemicals were of the highest quality available.

### 4.2. Animals

The protocols for the animal studies were approved by the Institute of Laboratory Animal Resources of Dongguk University_Seoul, Seoul, South Korea (IRB number: 2015-0044). Six-week-old male Sprague-Dawley (SD) rats were obtained from Charles River Orient (Seoul, South Korea). Upon arrival, rats were randomized and housed in groups of three per cage under strictly controlled environmental conditions at a temperature of 20–25 °C and 48–52% relative humidity for one week before the study. A 12 h light/dark cycle was used at an intensity of 150 to 300 lux. The rats were allowed free access to food and water before the experiment and then fasted with free access to water for 12 h.

### 4.3. Preparation of Stock Solutions, Plasma Samples and Quality Control Samples

Stock solutions of Rb1, Rb2, Rc, Rd, Re, and Rg1 were dissolved in methanol and those of R-Rg2, S-Rg2, R-Rg3, and S-Rg3 were dissolved in dimethyl sulfoxide, respectively, at 5 mg/mL. The stock solutions of 10 ginsenosides were serially diluted with methanol from 5 mg/mL to 1000, 500, 100, 50, 10, 5, 3, 2, 1, 0.5, 0.3, 0.2 and 0.1 µg/mL. The 1000, 500, 100, 50, 10, 5, 3, 2, and 1 µg/mL stock solutions of 10 ginsenosides were spiked with drug-free rat plasma to obtain final concentrations of 10, 5, 1, 0.5, 0.1, 0.05, 0.03, 0.02 and 0.01 µg/mL. To obtain quality control (QC) samples, stock solutions of each ginsenoside at 500, 50, 5 and 1 µg/mL were spiked into drug-free rat plasma to achieve final concentrations of 5 (high QC), 0.5 (medium QC), 0.05 (low QC), and 0.01 (lower limit of quantification, LLOQ) µg/mL as QC samples. The stock solution (2 mg/mL) of digoxin (IS) was prepared in methanol and further diluted in methanol to yield 0.5 µg/mL concentration for routine use as an IS.

### 4.4. Sample Preparations

A 50 µL of plasma sample was deproteinized by adding 100 µL methanol containing 0.5 µg/mL of IS. After vortexing for 5 min and centrifugation for 10 min at 12,000 rpm and 4 °C, a 10 µL supernatant was injected into the UPLC-MS/MS system for analysis.

### 4.5. UPLC-MS/MS Conditions

All analyses were performed using a Waters UPLC-XEVO TQ-S system (Waters Corporation, Milford, MA, USA). The chromatographic separation was carried out using RP C18 column (ACQUITY UPLC BEH, 2.1 mm × 100 mm i.d., 1.7 µm particle size; Waters, Dublin, Ireland) at flow rate of 0.3 mL/min. The mobile phase was composed of 0.1% formic acid in water (A), acetonitrile (B) and methanol (C). The gradient elution was performed using the mobile phase comprising the following ratio of A: B: C with 100:0:0 (*v*/*v*/*v*) at time 0, 95:2.5:2.5 (*v*/*v*/*v*) to 3 min, 76.1:11.95:11.95 (*v*/*v*/*v*) to 5 min, 26.1:36.95:36.95 (*v*/*v*/*v*) to 20 min, 26.1:40.6:33.3 (*v*/*v*/*v*) to 20.1 min and 100:0:0 (*v*/*v*/*v*) to 28.5 min with a linear gradient at each interval. Further, A: B: C at a ratio of 100:0:0 (*v*/*v*/*v*) was maintained until 30 min. The total run time was 30 min.

**Figure 4:**
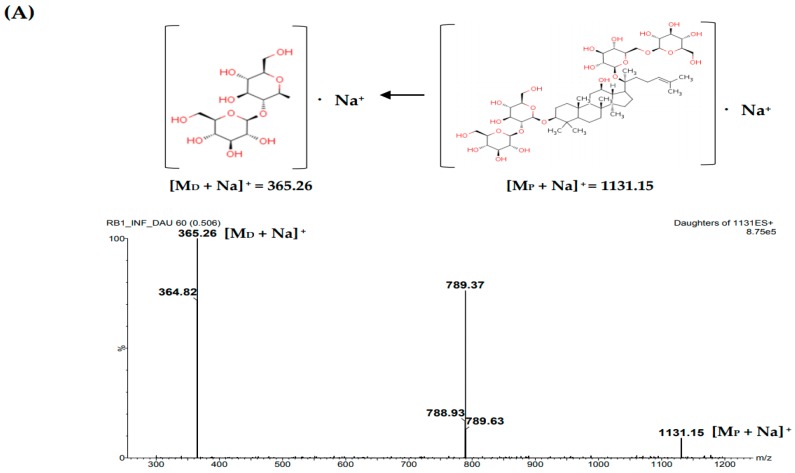

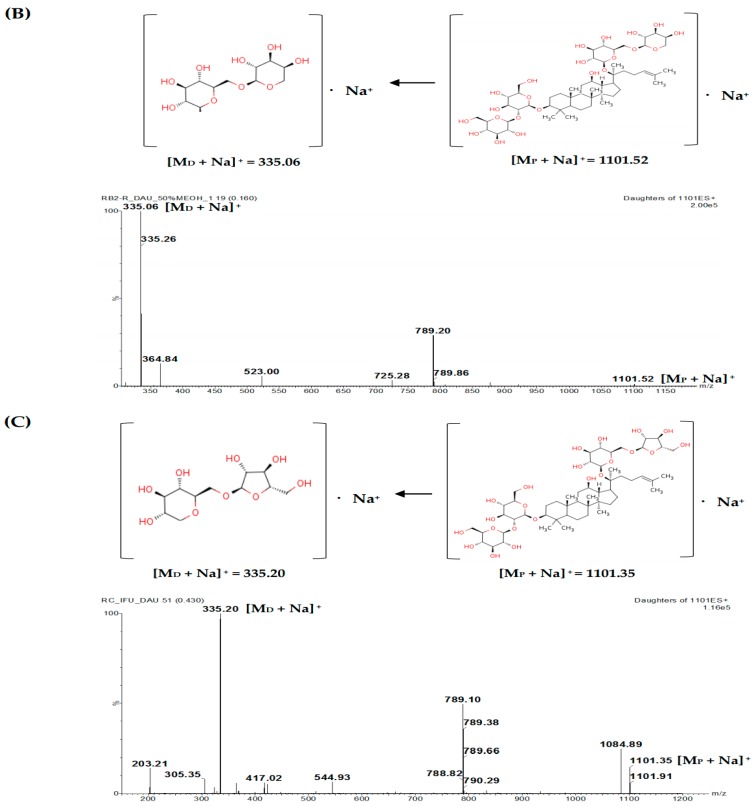

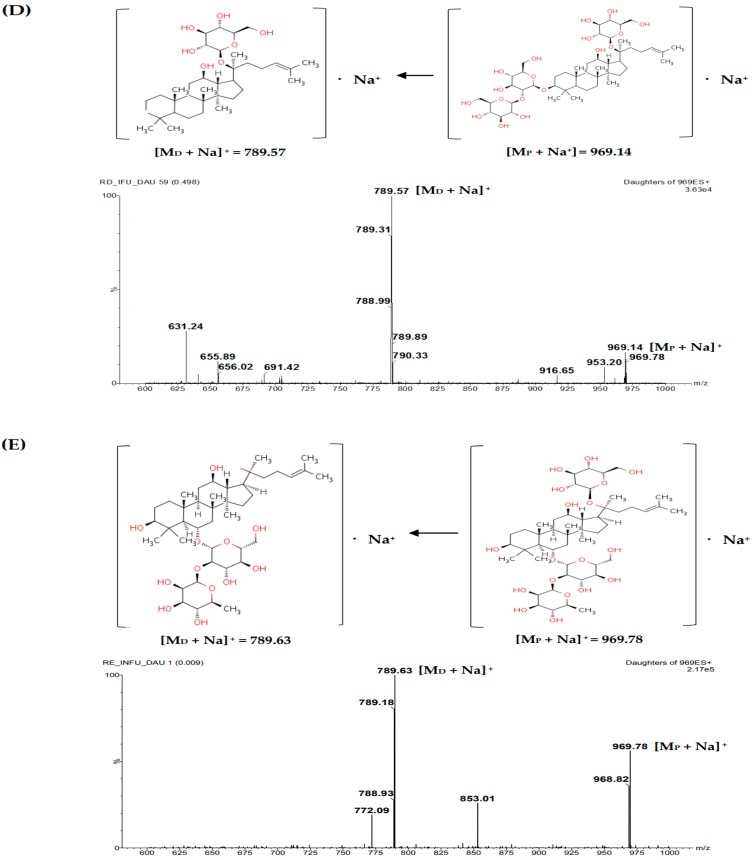

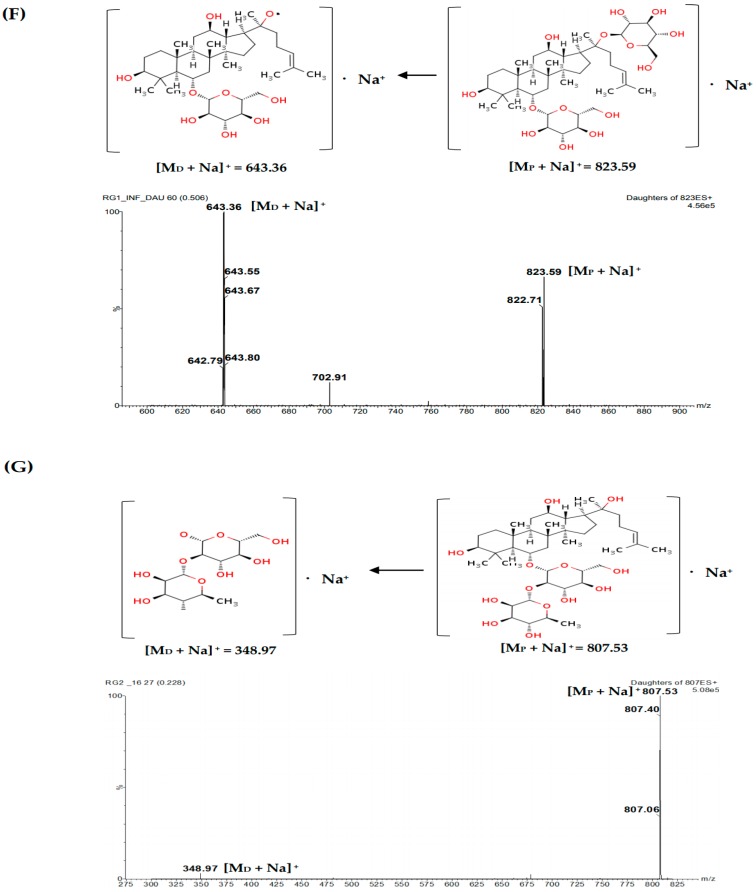

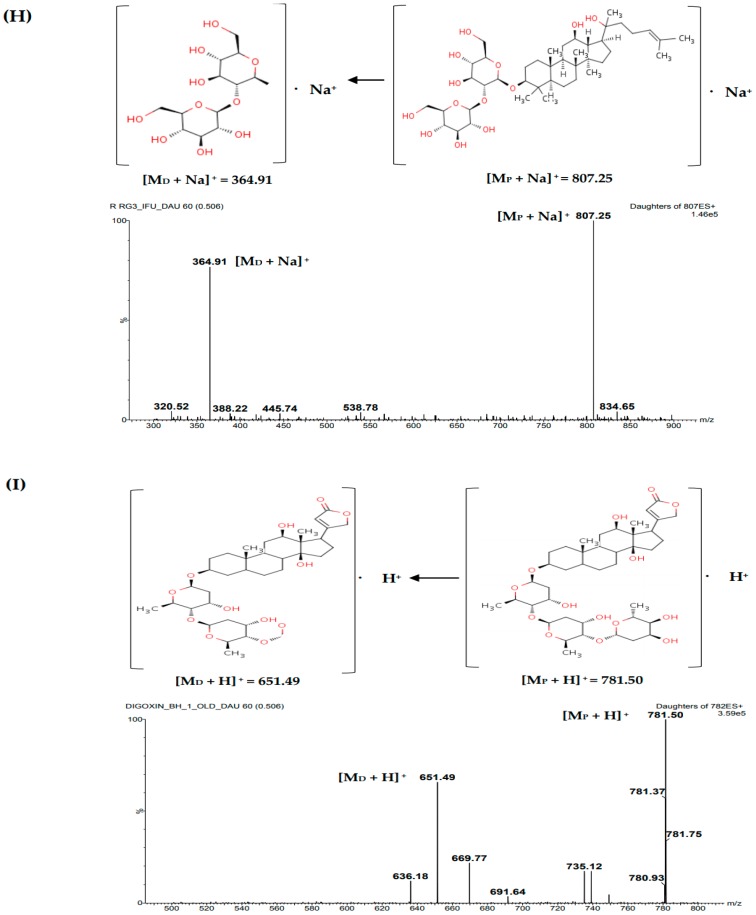
MS/MS spectra and proposed fragmentations for (**A**) Rb1, (**B**) Rb2, (**C**) Rc, (**D**) Rd, (**E**) Re, (**F**) Rg1, (**G**) Rg2, (**H**) Rg3, and (**I**) IS.

The multiple reaction monitoring (MRM) mode with electrospray ionization (ESI) interface was used for positive ions ([M + Na]^+^) and ([M + H]^+^) for ginsenosides and IS respectively at a capillary voltage of 3.0 kV, a source temperature of 650 °C and desolvation gas temperature of 350 °C. The *m*/*z* values for Rb1, Rb2, Rc, Rd, Re, Rg1, R-Rg2, S-Rg2, R-Rg3, S-Rg3, and IS were 1131.15→365.26 (80 and 65 eV for cone voltage and collision energy, respectively), 1101.52→335.06 (CV 90, CE 65), 1101.35→335.20 (CV 95, CE 50), 969.14→789.57 (CV 90, CE 35), 969.78→789.63 (CV 90, CE 45), 823.59→643.36 (CV 80, CE 40), 807.53→348.97 (CV 80, CE 50), 807.53→348.97 (CV 80, CE 50), 807.25→364.91 (CV 90, CE 40), 807.25→364.91 (CV 90, CE 40), and 781.50→651.49 (CV 25, CE 10), respectively, as shown in Figure 4. The analytical data were processed using MassLynx software (Version 4.1, Waters Corporation, Ireland).

### 4.6. UPLS-MS/MS Analytical Validation Assays

UPLC-MS/MS assays for analytical validation were conducted considering the bioanalytical method validation procedure currently accepted by United States Food and Drug Administration (FDA guideline, 2018). The validation parameters consist of selectivity, linearity, sensitivity, accuracy, precision, and stability of Rb1, Rb2, Rc, Rd, Re, Rg1, R-Rg2, S-Rg2, R-Rg3, and S-Rg3 in rat plasma samples.

Selectivity was evaluated by comparing the chromatograms of six different batches of plasma obtained from six rats to ensure the absence of interfering peaks at the respective retention times of Rb1, Rb2, Rc, Rd, Re, Rg1, R-Rg2, S-Rg2, R-Rg3, and S-Rg3 at LLOQ levels. 

Linearity of each matching calibration curve was determined by plotting the peak area ratio (y) of Rb1, Rb2, Rc, Rd, Re, Rg1, R-Rg2, S-Rg2, R-Rg3, and S-Rg3 relative to the IS area versus the nominal concentration (x) of each ginsenoside. The calibration curves were constructed by a weighting factor with a mean linear regression equation, y = ax + b. The LLOQ is defined as the lowest concentration of analytes yielding an S/N of at least 10. 

Intra- and inter-day accuracy and precision were determined by analyzing six replicates of the LLOQ sample and three different QC samples on five different days. The accuracy and precision was expressed by the following equations. The concentrations of LLOQ and QC samples were determined based on the standard calibration curve and analyzed on the same day.

The accuracy was expressed as:

$$\mathrm{Accuracy}\left(\%\right)=\frac{mean\;observed\;concentration}{nominal\;concentration}\times 100$$

The precision was expressed as the relative standard variation (RSD):

$$\mathrm{RSD}\left(\%\right)=\frac{standard\;deviation}{mean\;concentration}\times 100$$

Matrix effect was calculated by the following equation using the peak analyte areas obtained by direct injection of diluted (or neat) standard solutions (A) and the corresponding peak areas of diluted (or neat) standard solutions spiked into plasma deprotenized acetonitrile (B). The final analyte concentrations used to calculate the matrix effect were similar to QC sample levels: 0.05, 0.5 and 5 µg/mL. Also, the matrix effect of IS was 0.5 µg/mL. Further, the matrix effect of IS (0.5 µg/mL) was evaluated using the same method.

$$\mathrm{Matrix}\;\mathrm{effect}\;\left(\%\right)=\frac{B}{A}\times 100$$

Stability was assessed at 0.05, 0.5, and 5 µg/mL by analyzing samples in triplicate after four different manipulations: short-term storage (room temperature for 24 h), three-thaw cycles, post-treatment storage (24 h at 4 °C), and long-term storage (28 days at −20 °C).

### 4.7. Pharmacokinetic Study in Rats

To verify the pharmacokinetic applications of the analytical method developed, a pharmacokinetic investigation of Rb1, Rb2, Rc, Rd, Re, Rg1, R-Rg2, S-Rg2, R-Rg3, and S-Rg3 after oral administration of 600 mg/kg GBE in rats was conducted. On the experimental day, the carotid artery was cannulated in 6-week-old male SD rats as described previously [34]. After recovery from anesthesia, a 600 mg (5 mL)/kg of GBE dissolved in saline was orally administered to rats. A 0.12 mL blood sample was collected via the carotid artery at 0, 5, 15, 30, 45, 60, 90, 120, 180, 240, 360, 480, 600, 720, 1200, 1440, 1620 and 1800 min after oral administration of GBE. Plasma samples of 50 µL were obtained by centrifugation of each blood sample at 9000 rpm for 1 min and then stored at −20 °C for analysis of Rb1, Rb2, Rc, Rd, Re, Rg1, R-Rg2, S-Rg2, R-Rg3, and S-Rg3. Standard methods [35] were used to calculate the pharmacokinetic parameters using a non-compartmental analysis (WinNonlin 2.1; Pharsight Corp., Mountain View, CA, USA). The peak plasma concentration (*C*_max_) and time to reach *C*_max_ (*T*_max_) were determined directly from the experimental data.

## Figures and Tables

**Table 1:** The regression equations, linear ranges, and lower limits of quantification (LLOQs) in rat plasma.

Analytes	Regression Equation	*r* ^2^	Linear Range (µg/mL)	LLOQ (µg/mL)
Rb1	y = 0.1661x + 0.000064	0.9999	0.01–10	0.01
Rb2	y = 0.2190x − 0.00010	0.9999	0.01–10	0.01
Rc	y = 0.2850x − 0.00040	0.9993	0.01–10	0.01
Rd	y = 0.2185x + 0.00062	0.9993	0.01–10	0.01
Re	y = 0.07290x + 0.0027	0.9987	0.01–10	0.01
Rg1	y = 0.1432x + 0.021	0.9824	0.01–10	0.01
R-Rg2	y = 0.05100x + 0.0026	0.9978	0.01–10	0.01
S-Rg2	y = 0.05420x + 0.0012	0.9996	0.01–10	0.01
R-Rg3	y = 0.1792x − 0.00020	1.000	0.01–10	0.01
S-Rg3	y = 0.1285x + 0.0006	1.000	0.01–10	0.01

**Table 2:** Intra- and inter-day precision and accuracy for the determination of ten ginsenosides in rat plasma samples.

Spiked Concentration (µg/mL)	Intra-Day			Inter-Day		
	Precision		Accuracy (%)	Precision		Accuracy (%)
	Mean ± SD	RSD ^a)^ (%)		Mean ± SD	RSD ^a)^ (%)	
Rb1						
0.01	0.161 ± 0.0080	4.78	95.0	0.164 ± 0.0067	4.09	97.0
0.05	0.166 ± 0.0040	2.44	99.0	0.168 ± 0.00090	0.543	99.5
0.5	0.164 ± 0.0080	4.86	98.7	0.169 ± 0.0018	1.05	100
5	0.167 ± 0.0011	0.664	101	0.167 ± 0.00030	0.175	99.1
Rb2						
0.01	0.211 ± 0.0056	2.64	98.9	0.217 ± 0.0037	1.72	109
0.05	0.218 ± 0.0058	2.64	101	0.220 ± 0.0021	0.970	104
0.5	0.216 ± 0.0033	1.54	98.7	0.217 ± 0.0028	1.30	98.6
5	0.221 ± 0.0078	3.54	101	0.223 ± 0.0047	2.09	101
Rc						
0.01	0.250 ± 0.023	9.20	95.0	0.283 ± 0.0096	3.40	111
0.05	0.272 ± 0.012	4.27	98.3	0.278 ± 0.014	4.99	102
0.5	0.289 ± 0.032	11.0	102	0.276 ± 0.015	5.28	98.1
5	0.278 ± 0.010	3.63	97.4	0.284 ± 0.0085	2.99	100
Rd						
0.01	0.237 ± 0.017	6.99	94.3	0.243 ± 0.025	10.2	98.9
0.05	0.223 ± 0.0080	3.61	96.3	0.220 ± 0.0040	1.82	95.1
0.5	0.213 ± 0.0069	3.25	97.1	0.218 ± 0.0066	3.04	97.8
5	0.213 ± 0.0079	3.71	97.3	0.218 ± 0.0050	2.29	98.3
Re						
0.01	0.0791 ± 0.00019	0.0237	102	0.0777 ± 0.011	14.9	86.0
0.05	0.0794 ± 0.00052	0.659	101	0.0797 ± 0.0055	6.88	96.5
0.5	0.0784 ± 0.00075	0.959	98.8	0.0818 ± 0.0052	6.31	100
5	0.0739 ± 0.0025	3.41	93.1	0.0796 ± 0.0069	8.78	90.4
Rg1						
0.01	0.196 ± 0.0034	1.74	102	0.188 ± 0.0203	10.8	94.3
0.05	0.196 ± 0.0011	0.570	101	0.189 ± 0.012	6.36	98.4
0.5	0.196 ± 0.0029	1.47	100	0.193 ± 0.013	6.85	101
5	0.195 ± 0.0065	3.39	93.9	0.192 ± 0.0122	6.51	96.2
R-Rg2						
0.01	0.0579 ± 0.0028	4.91	103	0.0589 ± 0.0020	3.47	103
0.05	0.0585 ± 0.0026	4.41	103	0.0585 ± 0.00046	0.793	102
0.5	0.0568 ± 0.0010	1.80	98.6	0.0572 ± 0.00121	2.05	99.2
5	0.0508 ± 0.0011	2.16	88.1	0.0562 ± 0.00162	2.97	88.3
S-Rg2						
0.01	0.0574 ± 0.0045	7.79	89.7	0.0618 ± 0.00068	1.10	110
0.05	0.0594 ± 0.0038	6.34	99.8	0.0578 ± 0.0021	3.60	99.4
0.5	0.0584 ± 0.0013	2.25	102	0.0600 ± 0.00066	1.10	101
5	0.0542 ± 0.0038	7.03	94.9	0.0539 ± 0.0018	3.39	90.4
R-Rg3						
0.01	0.179 ± 0.0022	11.5	105	0.172 ± 0.016	9.07	89.3
0.05	0.179 ± 0.00061	0.342	102	0.178 ± 0.00092	0.519	95.2
0.5	0.179 ± 0.0016	0.876	100	0.177 ± 0.00072	0.408	99.4
5	0.178 ± 0.0012	0.686	99.5	0.177 ± 0.0026	1.48	99.8
S-Rg3						
0.01	0.144 ± 0.0152	10.8	107	0.122 ± 0.0123	10.4	97.6
0.05	0.130 ± 0.00079	0.610	95.6	0.129 ± 0.00061	0.474	98.3
0.5	0.130 ± 0.0034	2.65	100	0.130 ± 0.0029	2.21	101
5	0.128 ± 0.0013	1.01	99.5	0.129 ± 0.00074	0.572	100

^a)^ RSD, relative standard variation (SD/mean × 100).

**Table 3:** Mean recovery values (%) of stability study in rat plasma samples under various conditions.

Spiked Concentration (µg/mL)		Short-Term Storage (25 °C)	Three-Thaw Cycles	Post-Treatment (25 °C)	Long-Term Storage (−80 °C)
**Rb1**	0.05	109	96.9	88.2	98.1
	0.5	97.4	91.4	93.6	99.6
	5	103	103.5	95.3	103
**Rb2**	0.05	97.5	97.3	94.6	95.4
	0.5	93.6	94.5	94.9	101
	5	93.6	102	99.5	107
**Rc**	0.05	109	102	107	90.8
	0.5	107	103	100	97.9
	5	105	106	92.2	103
**Rd**	0.05	96.2	93.5	74.3	88.7
	0.5	100	94.4	78.4	99.0
	5	96.3	94.1	72.9	93.0
**Re**	0.05	104	109	102	98.6
	0.5	107	99.3	106	97.3
	5	99.8	102	109	94.8
**Rg1**	0.05	98.5	104	103	87.3
	0.5	97.2	104	109	91.0
	5	97.6	104	101	95.0
**R-Rg2**	0.05	103	101	96.1	92.9
	0.5	98.9	97.3	90.4	90.5
	5	105	108	95.5	94.0
**S-Rg2**	0.05	99.6	103	96.6	97.9
	0.5	110	101	94.1	93.5
	5	108	101	95.9	101
**R-Rg3**	0.05	104	109	103	99.7
	0.5	101	106	107	95.5
	5	103	109	101	106
**S-Rg3**	0.05	95.8	105	108	108
	0.5	97.8	107	105	93.4
	5	96.1	101	101	102

**Table 4:** Plasma concentrations of ginsenosides after oral administration of 600 mg/kg GBE to SD rats.

	**Rb1**			**Rb2**			**Rc**		
AUC_last_ (µg·min/mL)	202	±	168	376	±	214	353	±	190
Normalized AUC_last_ (µg·min/mL)	26.1	±	21.7	24.5	±	13.9	68.5	±	36.9
*C*_max_ (µg/mL)	0.210	±	0.183	0.395	±	0.285	0.387	±	0.292
Normalized *C*_max_ (µg/mL)	0.0272	±	0.0237	0.0257	±	0.0185	0.0751	±	0.0566
*T*_max_ (h)	8 (4–12)			10 (8–12)			10 (8–12)		
t_1/2_ (h)	16.5	±	2.71	15.9	±	1.35	14.9	±	1.84
	**Rd**			**Re**			**Rg1**		
AUC_last_ (µg·min/mL)	543	±	384	349	±	68.0	22.8	±	5.83
Normalized AUC_last_ (µg·min/mL)	27.3	±	19.3	8.96	±	1.74	15.8	±	4.05
*C*_max_ (µg/mL)	0.519	±	0.293	3.32	±	4.74	0.225	±	0.216
Normalized *C*_max_ (µg/mL)	0.0261	±	0.0148	0.0852	±	0.121	0.156	±	0.150
*T*_max_ (h)	480 (15–600)			360 (5–720)			3 (0.083–6)		
t_1/2_ (h)	12.9	±	1.28	10.8	±	5.73	10.3	±	3.11
	**S-Rg2**			**R-Rg2**			**S-Rg3**		
AUC_last_ (µg·min/mL)	9.98	±	3.36	4.21	±	1.02	3.57	±	2.03
Normalized AUC_last_ (µg·min/mL)	2.79	±	1.66	1.49	±	1.02	4.25	±	2.42
*C*_max_ (µg/mL)	0.284	±	0.163	0.108	±	0.0153	0.0470	±	0.0537
Normalized *C*_max_ (µg/mL)	0.0777	±	0.0495	0.0383	±	0.00452	0.0560	±	0.0640
*T*_max_ (h)	0.25 (0.083–0.25)			0.25 (0.083–0.25)			1 (0.25–1.5)		
t_1/2_ (h)	2.38	±	1.67	1.54	±	0.353	3.12	±	1.22
